# Recruitment Drives Spatial Variation in Recovery Rates of Resilient Coral Reefs

**DOI:** 10.1038/s41598-018-25414-8

**Published:** 2018-05-09

**Authors:** Sally J. Holbrook, Thomas C. Adam, Peter J. Edmunds, Russell J. Schmitt, Robert C. Carpenter, Andrew J. Brooks, Hunter S. Lenihan, Cheryl J. Briggs

**Affiliations:** 1 Department of Ecology, Evolution and Marine Biology, University of California Santa Barbara, Santa Barbara, CA 93106 USA; 2 Coastal Research Center, Marine Science Institute, University of California Santa Barbara, Santa Barbara, CA 93106 USA; 3 Department of Biology, California State University Northridge, Northridge, CA 91330 USA; 4 Bren School of Environmental Science and Management, University of California Santa Barbara, Santa Barbara, CA 93106 USA

## Abstract

Tropical reefs often undergo acute disturbances that result in landscape-scale loss of coral. Due to increasing threats to coral reefs from climate change and anthropogenic perturbations, it is critical to understand mechanisms that drive recovery of these ecosystems. We explored this issue on the fore reef of Moorea, French Polynesia, following a crown-of-thorns seastar outbreak and cyclone that dramatically reduced cover of coral. During the five-years following the disturbances, the rate of re-establishment of coral cover differed systematically around the triangular-shaped island; coral cover returned most rapidly at sites where the least amount of live coral remained after the disturbances. Although sites differed greatly in the rate of return of coral, all showed at least some evidence of re-assembly to their pre-disturbance community structure in terms of relative abundance of coral taxa and other benthic space holders. The primary driver of spatial variation in recovery was recruitment of sexually-produced corals; subsequent growth and survivorship were less important in shaping the spatial pattern. Our findings suggest that, although the coral community has been resilient, some areas are unlikely to attain the coral cover and taxonomic structure they had prior to the most recent disturbances before the advent of another landscape-scale perturbation.

## Introduction

Tropical coral reefs are subject to a variety of disturbances such as cyclones, bleaching events, and outbreaks of predators that all can result in landscape-scale loss of coral cover and dramatic alteration of the entire reef community. Reefs can be resilient to these disturbances either by resisting change or by rapidly recovering to their pre-disturbed state. Thus, one component of resilience is the rate of recovery of a disturbed reef following coral decline. Several decades of research have explored the causes of declining coral cover, as well as patterns of community responses and the processes affecting the rate of re-establishment of a coral-dominated community. In an early study, Connell^1^ surveyed three decades of literature to assess the frequency of declines in cover of coral and whether - and to what degree - there was subsequent return of coral cover to pre-disturbance values. He argued that the potential for recovery was related to the disturbance type, with acute, short-term disturbances more likely (69%) to be followed by recovery than chronic, long-term disturbances (27%). For example, at Heron Island on the Great Barrier Reef, complex patterns of coral loss and recovery among four different habitats depended on the duration, frequency, and type of disturbance^2^. Further, the time scales of recovery of coral cover to pre-disturbance values after acute disturbances (i.e., cyclones) has varied from around a decade in areas where the substratum was not greatly damaged (i.e., physically crushed and broken) by the disturbance, to two decades where disturbances greatly altered physical structure^2,3^.

The propensity for coral communities to recover from perturbations on Indo-Pacific reefs contrasts sharply with the Caribbean, where corals have generally failed to recover from disturbances since at least the early 1980’s, and there has been a widespread pattern of shifts from coral- to a macroalgae-dominated state in many locations^4–6^. Following worldwide coral bleaching in 1997/98, in which coral mortality was high, several Indo-Pacific reefs underwent rapid recovery^7–10^. On a regional scale, assessment of changes in coral cover following acute disturbances in 48 locations across the Indo-Pacific revealed the rate of return to the pre-disturbed level was related to a variety of factors including region (Eastern Pacific slowest, western Pacific fastest), management status, and severity of disturbance^8^. Comparative studies of coral community dynamics in select locations identified several mechanisms that influenced the rate of coral recovery, with patterns of recovery driven by differences in the demographic rates of different coral taxa, including recruitment, post-settlement growth and survival, and the capacity for regrowth of remnant colonies^7,9,11^. These kinds of studies are critical to understanding why coral reefs differ in their ability to recover from disturbances. However, they are rare in coral reef literature because reefs are often sampled irregularly, and many of the biological and physical variables necessary to test the importance of different mechanisms in determining the rate of population recovery for multiple coral taxa are not measured. Additional studies are needed to better understand mechanisms underlying spatial and temporal variation in coral recovery following disturbances^8^.

Although studies of the effects of disturbances on coral reefs have largely focused on a single benthic state variable (i.e., coral cover), there has been increasing interest in quantifying other aspects of coral community ‘recovery’ following disturbances. A key issue associated with analyzing recovery of a reef community following disturbance is the extent to which the coral assemblage and associated biota (e.g., fishes, macroalgae, non-coral invertebrates) re-assemble to the pre-disturbance state^3,11–18^. Understanding the factors determining the similarity of pre- and post- disturbance communities is central to our understanding of community resilience, because coral reefs can recover to pre-disturbance levels of coral cover but shift in the assemblage structure of corals and associated biota^3,12,19^.

The fore reef of Moorea, French Polynesia, has undergone repeated disturbances over the past several decades that have resulted in landscape-scale loss of coral followed by return to pre-disturbance cover within about a decade^12,20–25^. These have included cyclones, coral bleaching events, and outbreaks of predatory crown-of-thorns sea stars. The most recent major disturbances, a crown-of-thorns sea star outbreak that peaked between 2007 and 2009 and a cyclone (2010), dramatically reduced cover of live coral on the fore reef^23,26^, affording the opportunity to quantify patterns of recovery for two attributes of the coral community (coral cover and community re-assembly) around the island and to explore the underlying mechanisms driving observed spatial patterns^27,28^. Here we address three major questions. First, is there variation in resilience of the fore reef coral community among the three shores of Moorea as measured by the spatial pattern of loss and return rate of live coral? Second, what demographic mechanisms underlie spatial variation in speed of recovery of coral cover? And third, is the benthic community – defined by cover of scleractinians, *Millepora*, and the abundance of other sessile benthic taxa – re-assembling to its pre-disturbance community structure?

## Results

### Spatial and temporal variation in the cover of live coral

**Figure 1:**
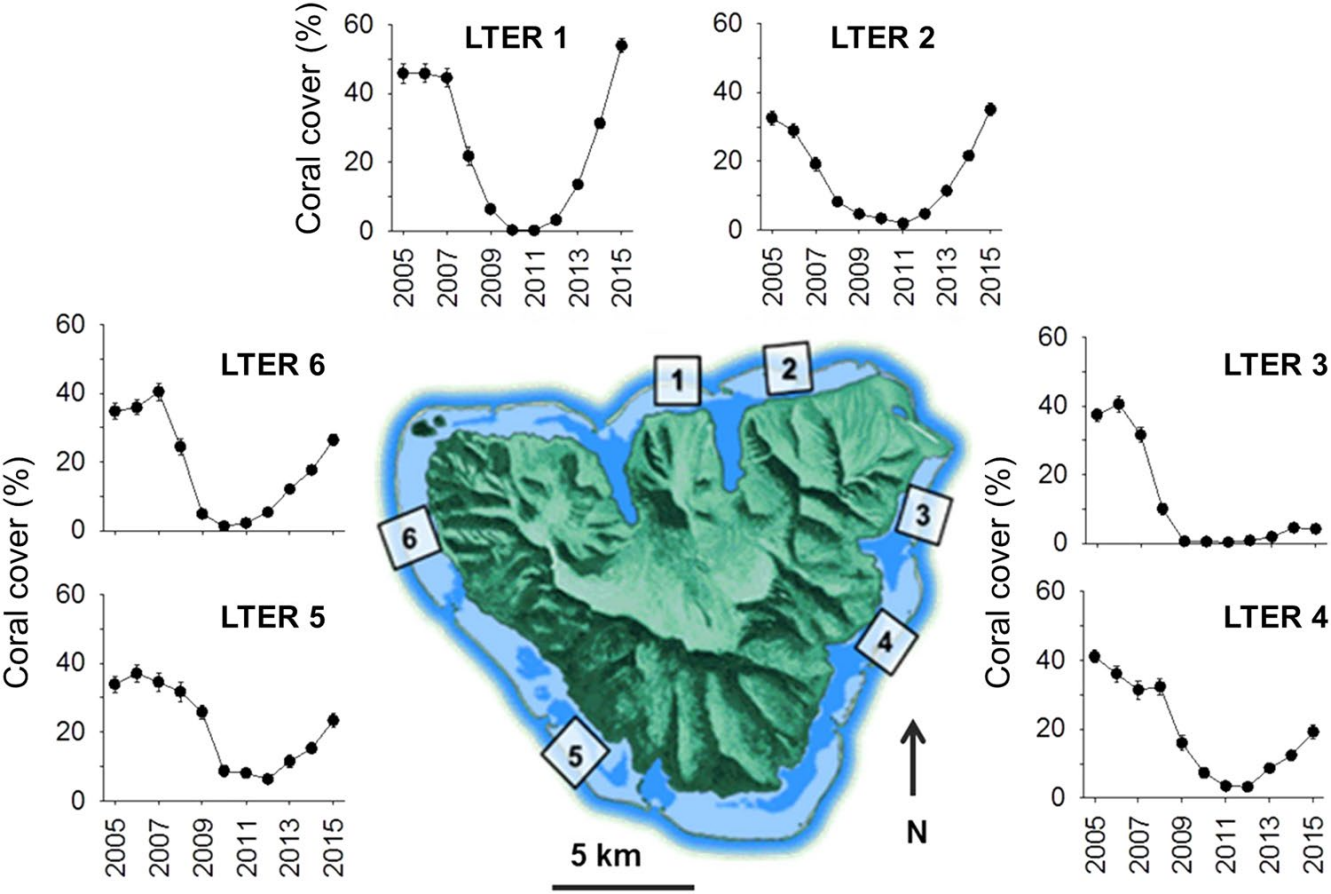
Spatial variation in mean (±SE) percent cover of live coral at 10 m depth at 6 fore reef sites around Moorea (n ~ 40 quadrats year^−1^ site^−1^), showing: (1) almost complete loss of coral cover at all 6 sites, and (2) strong spatial variation in rate of return of coral cover. Map of Moorea was based on an original image (ISS006-E-39837) provided courtesy of the Earth Science and Remote Sensing Unit, NASA Johnson Space Center (https://eol.jsc.nasa.gov) and was modified using Adobe Photoshop Elements v14.1 and Microsoft PowerPoint 2016.

The outbreak of crown-of-thorns sea stars (2007–09) followed by Cyclone Oli in February 2010 resulted in nearly complete loss of live coral on the fore reef^23,24,27^. Time series data from the six sites around Moorea revealed that the cover of live coral declined by ~90%, from ~40% in 2005 to <5% in 2010 (Fig. 1). There was variation in the timing and extent of the decline of coral. Most quadrats at sites on the north shore and northern ends of the east and west shores (1, 2, 3, and 6) reached their nadir of coral cover in 2010 or 2011, while most quadrats at the southern sites of the east and west shores (4 and 5) reached their nadir of coral cover in 2011 or 2012 (Supplementary Fig. S1). Although all sites had a positive trajectory of coral cover from 2011 to 2015, recovery rates differed among sites and ranged from ~1% y^−1^ to 12% y^−1^ (ANOVA, F_5,225_ = 69.32, P < 0.0001). The most rapid recovery occurred on the north shore, and the least rapid on the east shore (Fig. 1). Within five years of Cyclone Oli, the two north shore sites (LTER 1 and LTER 2) had exceeded their pre-disturbance cover of coral, in contrast to a site (LTER 3) on the east shore that had barely begun to increase in coral cover. The time-averaged cover of macroalgae during the recovery period remained ≤15% on all of the sites^23,24^, and an ANOVA that tested the significance of the regression model of the relationship between the rate of increase in coral cover and the cover of macroalgae during the post-disturbance period was not significant (F_1,4_ = 3.34, P = 0.14) (Fig. S2). The island wide percent cover of the most abundant genera of macroalgae during the post-disturbance period (averaged over all sites and years) was as follows: *Halimeda* – 5.0%, *Lobophora* – 1.5%, *Asparagopsis* – 1.2%, and *Turbinaria* – 1.0%.

Although many taxa of corals were present on the reefs, four genera – *Acropora*, *Montipora*, *Pocillopora*, and *Porites* – accounted for more than 80% of the live coral cover during our study (Supplementary Table S1). *Pocillopora* spp. were the most abundant group at all sites, ranging from 40% to 70% (by cover) of the total coral assemblage in 2015. As a result, rates of increase in coral cover were driven by rates of increase in *Pocillopora* spp. (Fig. S3; F_1,4_ = 88.56, P = 0.0007, r^2^ = 0.96). Thus we focused on *Pocillopora* spp. to explore demographic mechanisms underlying variation in rates of coral recovery.

### Demographic mechanisms underlying recovery of *Pocillopora*

**Figure 2:**
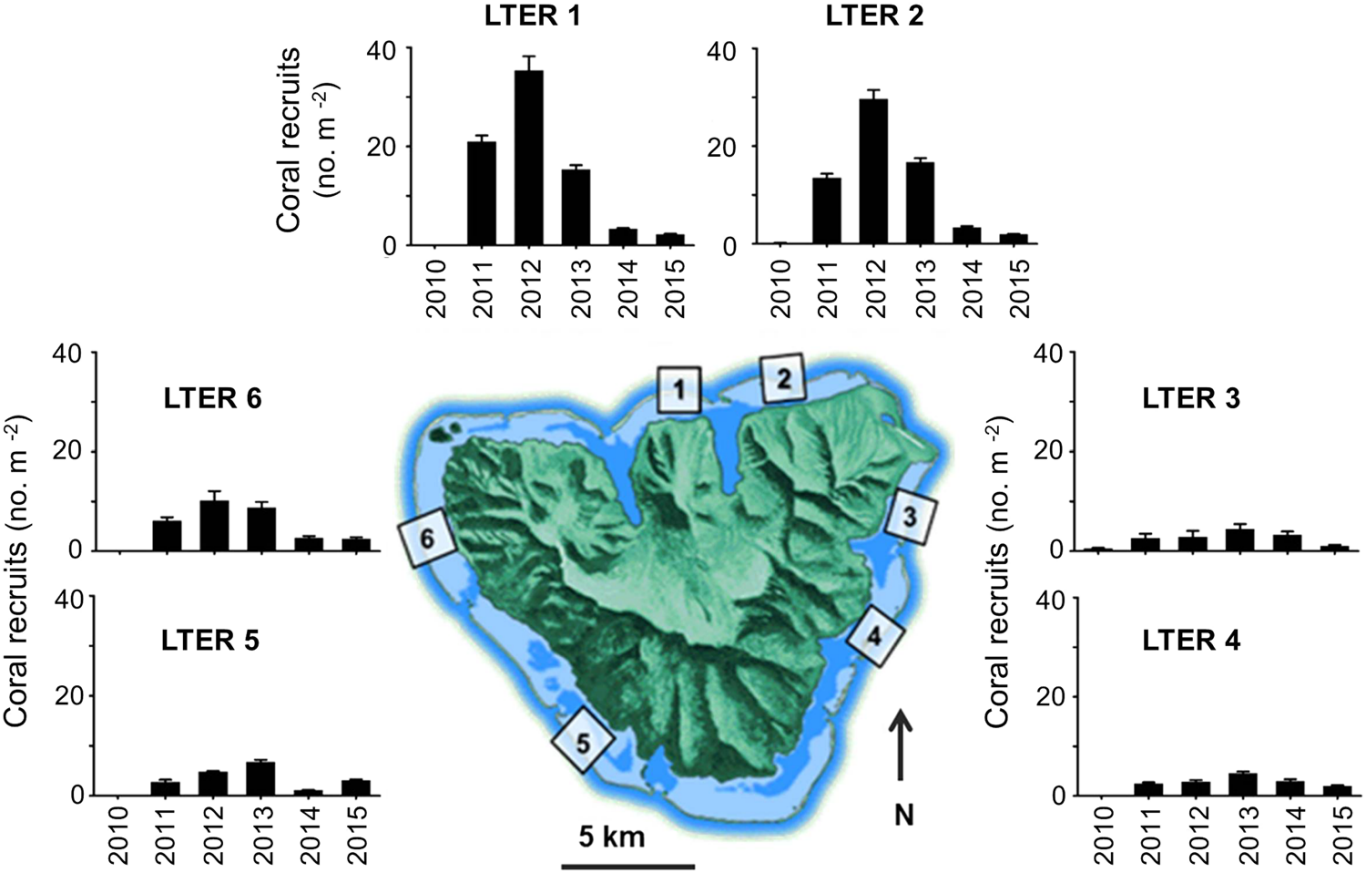
Spatial pattern in mean (±SE) density of coral recruits (colonies ≤3 cm diameter) at 10 m depth around Moorea based on diver surveys of 25 m^2^ plots. The magnitude of coral recruitment following 2010 varied sharply among sites, and the pattern mirrors the pattern of variation in recovery of live coral cover (Fig. 1). Map of Moorea was based on an original image (ISS006-E-39837) provided courtesy of the Earth Science and Remote Sensing Unit, NASA Johnson Space Center (https://eol.jsc.nasa.gov) and was modified using Adobe Photoshop Elements v14.1 and Microsoft PowerPoint 2016.

**Figure 3:**
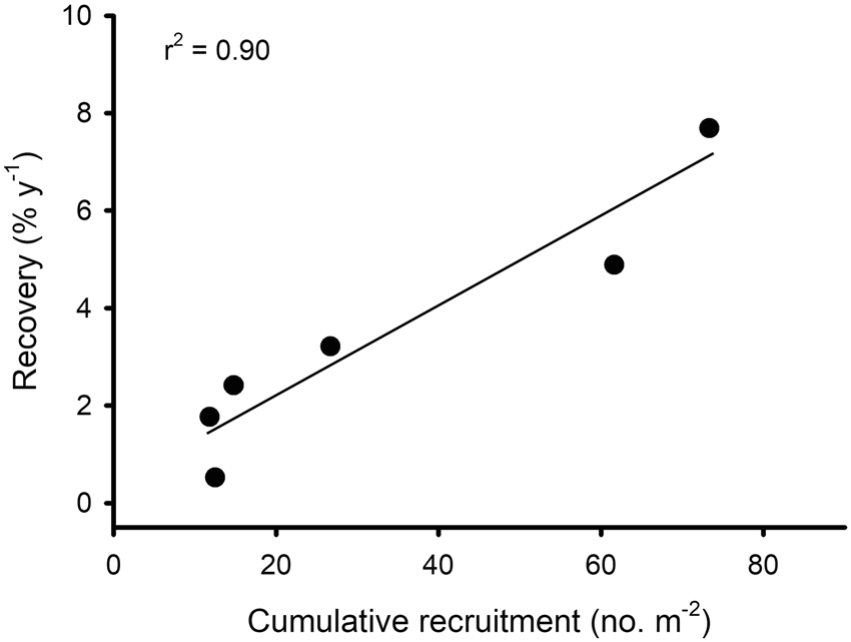
Relationship between the cumulative number of *Pocillopora* recruits per m^2^ from 2011 to 2014 and the rate of return of coral cover at 6 sites around Moorea. Line shows fit from least squares linear regression. N = 48,203 recruits observed during the time period at the six sites.

*In situ* diver surveys of sets of 25-m^2^ plots and analysis of 0.25-m^2^ photoquadrats from the six sites revealed strong spatial (among site) and temporal (among year) variation in *Pocillopora* recruitment (Fig. 2; Supplementary Fig. S4); the two estimates of recruitment were strongly positively correlated (Supplementary Fig. S5). Surveys of the 25-m^2^ plots revealed no recruits in August 2010, six months after Cyclone Oli, but by mid-2011, recruits were present at all sites, some at high densities (20 colonies m^−2^, Fig. 2). Despite strong among-year variation, there was a clear pattern of among-shore variation in coral recruitment, with most recruits on the north shore, fewer on the west shore, and the least on the east shore (Fig. 2, Supplementary Fig. S4). In each of the first three years following Cyclone Oli in 2010, mean densities of *Pocillopora* recruits ranged from 15 to 35 corals m^−2^ at the two sites on the north shore; during the same period recruit densities at the east shore sites were <5 *Pocillopora* corals m^−2^ (Fig. 2). Recruitment was consistent among 25-m^2^ diver-surveyed plots within a site, with site accounting for >80% of the variance in cumulative recruitment among plots (F_5,39_ = 41.92, P < 0.001). There was a significant positive relationship between site-level recovery rates and cumulative recruitment over the study period, with variation in recruitment among sites accounting for ~90% of the variation in recovery rates (F_1,4_ = 36.42, P = 0.004, r^2^ = 0.90) (Fig. 3). During the time period 2011–2014, a total of 48,203 recruits were observed in the diver surveys and 1,893 in the photoquadrats.

**Figure 4:**
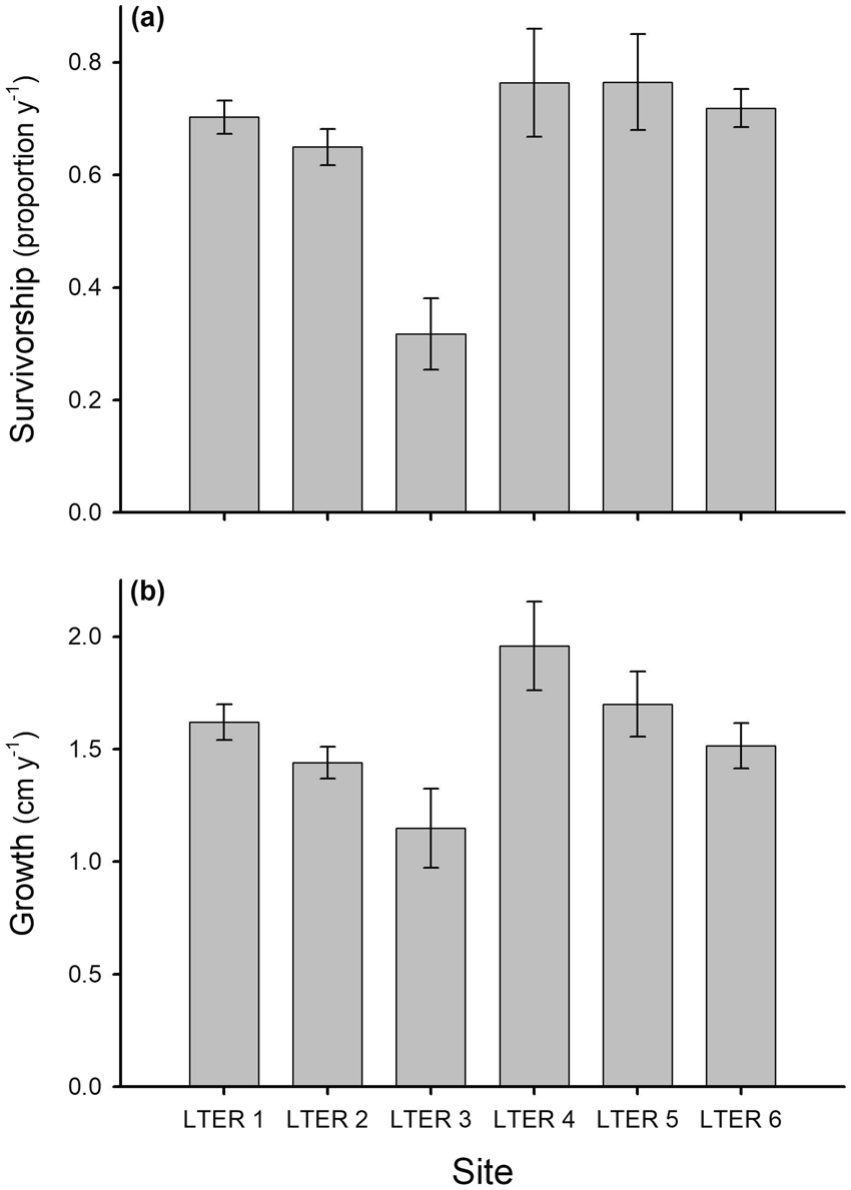
Variation among sites around Moorea in annual mean (±SE) survivorship (**a**) and mean (±SE) growth rates (**b**) of *Pocillopora* recruits. Variation in survivorship and growth did not match the spatial pattern in return of cover of live coral; these mechanisms appear insufficient to explain the observed variation among sites in return of coral.

In contrast to the among-site variation in *Pocillopora* recruitment, there was less among-site variation in their growth and survivorship. Year-to-year analyses of photoquadrats yielded sets of *Pocillopora* recruits that were tracked over time to estimate growth and survivorship (number of recruits - LTER 1: N = 518, LTER 2: N = 435, LTER 3: N = 100, LTER 4: N = 41, LTER 5: N = 37, LTER 6: N = 357). Only ~8% of the variance in mean growth of *Pocillopora* recruits could be attributed to site effects (F_5,148_ = 3.50, P = 0.005), and only ~20% of the variance in the mean annual survivorship of recruits could be attributed to site effects (F_5,173_ = 10.39, P < 0.001). Congruent with recruitment estimates from the 25-m^2^ diver plots, a large proportion (>50%) of the variance in the cumulative recruitment of *Pocillopora* detected in 0.25-m^2^ photoquadrats was attributed to site effects (F_5,202_ = 48.81, P < 0.001). Mean survivorship of recruits during their first year after detection in photoquadrats ranged from 32% at LTER 3 to 75% at LTER 5 (Fig. 4a), and mean (±SE) growth varied from 1.2 ± 0.2 cm y^−1^ at LTER 3 to 2.0 ± 0.2 cm y^−1^ at LTER 4 (Fig. 4b). Unlike coral recruitment, among-site variation in growth (F_1,4_ = 0.29, P = 0.62) and survivorship (F_1,4_ = 0.68, P = 0.56) of *Pocillopora* were not related to variation in rates of recovery.

In addition to the site-level analyses described above, we also tested whether the early demography of *Pocillopora* could explain variation in recovery at the 0.25-m^2^ photoquadrat scale. These analyses confirmed the patterns observed at the site scale. The model that best explained coral recovery on this small spatial scale included site, cumulative recruitment (total number of recruits from 2011 to 2014), minimum cover of *Pocillopora* spp. over the study (i.e., the nadir), and mean growth rates of recruits (Supplementary Table S2). This model explained ~62% of the variance in rate of coral recovery among photoquadrats, with >90% of the explainable variance accounted for by ‘site’ (F_5,140_ = 43.19, P < 0.0001, partial R^2^ = 0.40) and ‘cumulative recruitment’ (F_1,140_ = 19.60, P < 0.0001, partial R^2^ = 0.19). Average growth rates (i.e., mm y^−1^) of *Pocillopora* recruits were significantly and positively related to coral recovery rates (F_1,140_ = 4.37, P = 0.038), but their growth explained only a small fraction of the total variance in coral recovery rate in the final model (partial R^2^ = 0.02). Notably, coral recovery rates were negatively related to *Pocillopora* cover at the beginning of the recovery period (F_1,140_ = 7.55, P = 0.007), but this effect was less important than ‘site’ and ‘total recruitment’ (partial R^2^ = 0.03). The rates of coral recovery were unrelated to the average survivorship of *Pocillopora* recruits on a quadrat scale, and survivorship was not included in the final, best-fit model.

### Patterns of community re-assembly

**Figure 5:**
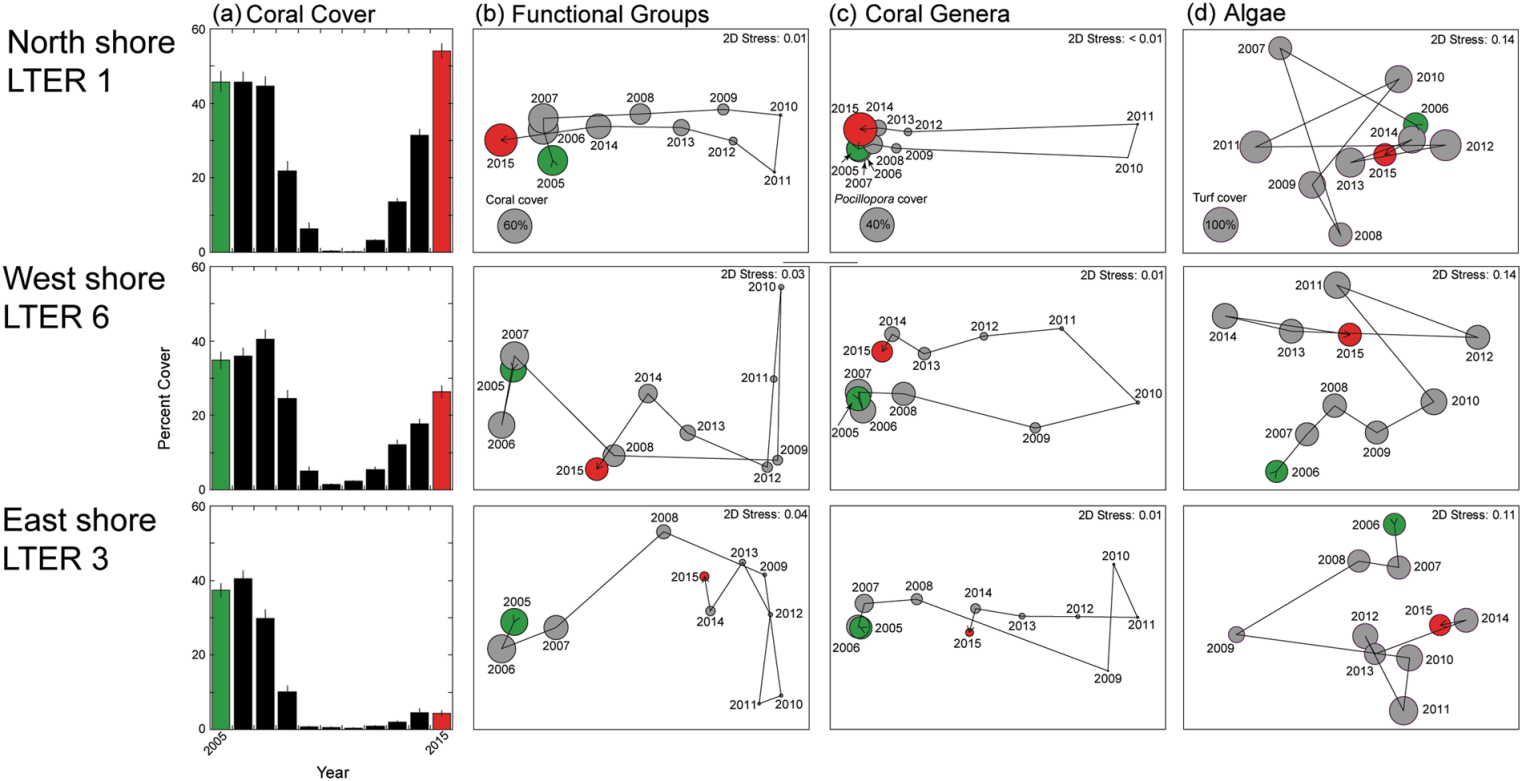
Variation in coral reef community structure between 2005 and 2015, during which corallivory by COTS and Cyclone Oli depressed coral cover close to zero by 2010. Variation in community structure is shown for one site on each side of the island (LTER 1 – north shore, LTER 6 – west shore, and LTER 3 – east shore) to illustrate spatial variation in rate of recovery. (**a**) Mean cover (±SE, n = 36–40 quadrats) of scleractinians and *Millepora* at the three sites. (**b**–**d**) NMDS plots based on analysis of community structure with (**b**) functional groups (**c**) coral taxa, and (**d**) algal taxa. Circles are scaled to show mean cover in each year of (**b**) scleractinians and *Millepora*, (**c**) *Pocillopora* spp. and (**d**) turf algae. Green = first year of analysis and red = last year of analysis.

**Figure 6:**
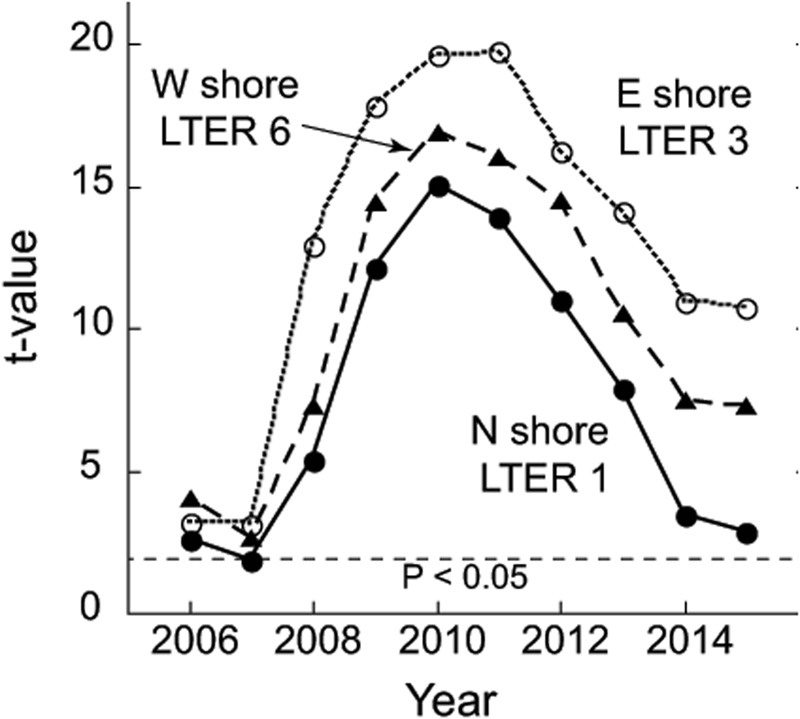
Results of post-hoc multiple contrasts (using t-tests, t values plotted) between multivariate community structure by functional group (see Fig. 5b) in 2005 versus the remaining 10 years for three sites. Analyses were conducted separately by site using PERMANOVA with years and time as factors in a repeated measures design with quadrats as replicates. Horizontal dashed line shows the critical value of t that identifies statistical significance (at P < 0.05).

The extent to which the broader benthic community recovered following the two disturbances was addressed using three aspects of benthic community structure: (1) the major benthic substratum categories, (2) taxonomic composition of corals, and (3) taxonomic composition of macroalgae. NMDS plots revealed a strong response in all three of these aspects to the disturbances, with trajectories indicating that recovery is occurring, as defined by the extent to which initial and final multivariate communities are similar (Fig. 5). From 2011 to 2015 (i.e., after the disturbances), benthic communities at 10 m depth on the outer reefs tracked towards their initial (i.e., in 2005) composition (Fig. 5). The rate at which sites re-assembled was related to their rates of return of coral cover, with north shore sites (e.g., LTER 1) qualitatively more similar (based on the close proximity of multivariate community states in 2-D ordination space) to their 2005 composition in all three aspects of benthic community structure than sites on either the west (e.g., LTER 6) or the east (e.g., LTER 3) shores (Fig. 6).

## Discussion

In the past several decades, there has been increasing interest in quantifying both the responses of coral reefs to disturbances that cause landscape-scale loss of corals, and to understand mechanisms that underlie spatial variation in ecosystem resilience^8,9,29,30^. Knowledge of these issues is fundamental to determining why some reefs transition to a largely non-coral state (i.e., macroalgae) following disturbances, while others regain a coral-dominated community^8–10,31–36^. In the Indo-Pacific, return of shallow coral reefs to pre-disturbance coral cover has been documented in multiple locations over the last several decades, but the speed of such recovery has varied among sites and depths, ranging from a few years to decades. For example, rapid return to coral dominance occurred following coral bleaching and mortality in 2006 on some inshore reefs of the Great Barrier Reef that drove coral cover from 77–89% to 20–30%, with several reefs regaining their pre-disturbance coral cover within a year^31^. Similarly, five years following a volcanic eruption that buried some reefs beneath lava flow on Gunung Api (Banda Islands, Indonesia) (i.e., coral cover was 0%), coral cover was >60%, and was higher than on nearby reefs that were unaffected by the eruption^32^. However, benthic communities on tropical reefs typically recovery more slowly, often taking a decade or more to return to their pre-disturbance coral cover. Studies from a diversity of locations including Guam^33^, Ryukyu Islands, Japan^34^, the Great Barrier Reef, Australia^3,35^, Moorea, French Polynesia^22^, Okinawa, Japan^36^, and Scott Reef, northwestern Australia^10^ have reported recovery from major disturbances in one to two decades.

Our descriptions of changing coral cover following recent major disturbances on Moorea reveal great variation in rate and extent of recovery among six fore reef sites, even though the sites are at the same depth (10 m), and by 2011 had experienced similar degree of reduction in live coral (Fig. 1). Within five years of the disturbances that reduced coral cover on the fore reef to near zero, two sites had fully regained – and almost exceeded - their pre-disturbance coral cover in 2005. By contrast, the rate of recovery of at least one site (on the north east shore) suggests full recovery of coral cover could require several decades, with the remaining sites falling between these extremes. Colgan^33^ argued that disturbances (such as bleaching or COTS outbreaks) that remove live coral but leave three-dimensional structure may result in faster community recovery than those that remove all major roughness elements from benthic surfaces. However, in the case of Moorea, the rate of return of coral cover appeared to be unrelated to how much structure remained on the benthos after the disturbances, as the north shore sites that exhibited the most rapid recovery (Fig. 1) had the least amount of physical structure (as well as the lowest amount of live coral) remaining following the disturbances^24^. This trend in variation of rate of recovery of coral cover was associated with the stronger impact of Cyclone Oli (February 2010) on the north shore of Moorea that cleared away virtually all of the dead coral skeletons that had previously been killed by the crown-of-thorns sea star outbreak^24^. The range of variation in recovery time reported here for the outer reef of Moorea brackets that reported for the Tiahura Transect on the fore reef of Moorea (~8 km west of LTER 1) following disturbances between 1991 and 2006. Adjeroud *et al*.^22^ noted that following disturbance and loss of coral in the early 1990’s, it took about a decade to return to pre-disturbance coral cover at that location.

Although spatio-temporal variation in coral cover is well-known throughout the Indo-Pacific and tropical west Atlantic, there is less information regarding the underlying mechanisms driving variation in recovery following acute disturbances. Potential demographic variables that could influence recovery include sexual recruitment, post-settlement growth and survival, regrowth of remnant colonies (including asexual proliferation), and growth of adult colonies^7,9,11^. However, time series studies often include limited information with which to evaluate the role of these demographic mechanisms in driving variation in coral recovery, in part because their focus is usually on describing changes in coral reef community structure over time.

Regrowth of remnant colonies that survive disturbances can play an important role in the initiation of recovery immediately after disturbances, especially when there is extensive partial mortality of individual coral colonies^37^. This phenomenon was critical in the rapid recovery from bleaching in the Keppel Islands, Australia, where coral cover was reduced from 77–89% to 20–30% as a result of bleaching^31^. Sites that showed the least recovery of coral communities had the lowest cover of coral immediately following the disturbance, as well as the least physical damage to the reef framework; the recruitment of sexual coral propagules was not a major factor promoting increases in coral cover at any site^31^. However, in most cases, recovery of coral communities following disturbances is a function of multiple processes including (but not limited to) coral recruitment, growth, and survival of new recruits, and the regrowth of remnant coral colonies or any remaining adults^7,10,11,38^. In Moorea following the most recent major disturbances, there was marked variation in rate of coral recovery around the island, which was driven primarily by differential rates of coral recruitment. Post-settlement growth and survival of coral recruits contributed little to variation in the return rate of coral cover. In addition, our results suggest that regrowth from remnants also did not play an important positive role, because few corals survived the crown-of-thorns outbreak and cyclone. Further, in contrast to the recent events on the Keppel Islands^31^, sites around Moorea that had the lowest cover of remnant live coral following the disturbances (i.e., sites on the north shore) experienced the highest recovery^24^, with coral cover 5 years after the disturbances equaling or exceeding coral cover 5 years prior to the events (Fig. 1).

Coral recruitment following the disturbance was highest on the north shore and tended to decrease in a counter clockwise direction around the island (Fig. 2). We do not know the mechanisms driving this gradient, but we hypothesize that local physical oceanographic processes involving features such as lagoon-fore reef circulation cells^39^ result in locally enhanced densities of pelagic coral larvae along the north shore of Moorea, with these larvae originating from coral parents located either in Moorea or nearby islands. It then is plausible that these concentrated patches of coral larvae are transported around the island by a shelf current that flows in a time-averaged counter-clockwise direction^39,40^. In this construct, coral larvae settle to benthic surfaces as the patches are transported around the island^41^ thereby creating a pattern consistent with patch depletion and downstream filtering^42–44^. The feasibility of this inferred mechanism is supported by the mean net velocity of surface water and the direction of the alongshore shelf current flow^40^, which together could transport a passive particle around the ~60 km perimeter of the island in ~21 days, as well as genetic studies of dispersal patterns of the anemonefish *Amphiprion chrysopterus*^45^. This model of larval depletion could help account for the paucity of coral settlers at LTER 3 and LTER 4 (Fig. 2). In addition, the steep profile and friable nature of the reef at LTER 3 may be contributing factors to the pattern of poorer growth and survival of young corals there.

Although the return of the outer reef at 10 m depth to pre-disturbance coral cover is one important metric of resilience of the coral community, full re-assembly of the coral community is also key to the restoration of the capacity to deliver essential goods and services^3^. The degree to which coral species composition and the relative abundance of individual coral taxa on reefs that have undergone cycles of disturbance and recovery have been altered is poorly understood^3,16,17,36,46^. However, taxonomic re-assembly of corals and other components of the reef community following disturbances typically varies among locations^3,10,12,17,47^. Johns *et al*.^17^ modeled trajectories and time frames of taxonomic re-assembly of corals at sites on the Great Barrier Reef, and predicted recovery of coral cover would occur within about a decade for five of the six sites examined. With respect to taxonomic re-assembly, four of the six were predicted to re-assemble to their pre-disturbance composition in less than 13 years^17^. On Moorea, we found a similar wide degree of variation in coral community re-assembly following disturbances, with these trends closely tracking the pace of re-establishment of coral cover. Our three metrics of benthic community structure – relative abundances of coral and macroalgal taxa as well as benthic functional groups – showed re-assembly to different degrees five years after disturbances, with the amount of re-assembly associated with the rate of return of coral cover. On the north shore, re-assembly to the pre-disturbance (2005) community composition was extensive by 2015, at least as evaluated by NMDS, whereas it had hardly begun on the east shore where the recovery of live coral was not extensive.

The high resilience of our two north shore sites with respect to cover of coral is consistent with temporal patterns of disturbance and recovery previously documented on the fore reef of the Tiahura transect on the north shore of Moorea since the late 1970’s. During this period, the fore reef of the Tiahura transect has undergone repeated disturbances and loss of coral cover due to cyclones, bleaching, and crown-of-thorns sea star outbreaks, but has returned to coral dominance within about a decade^12,22,25^. However, despite the strong tendency to regain coral cover rather than shift to dominance by algae or another benthic assemblage, there is evidence for a change in taxonomic composition of the fore reef coral community compared to the earliest period of modern ecological investigation in this location (i.e., the 1970’s). Critically, there has been weak population recovery of some *Acropora* spp. and increasing dominance of the community by *Pocillopora* spp. and *Porite*s spp.^12^. Because characteristics of the reef assemblages around Moorea, as well as disturbance regimes affecting them, are not well known prior to the 1970s, it is unclear whether the shifts in coral composition that took place in the decade(s) after the late 1970s reflect effects of directional climate change or a more recent increased frequency of disturbance that maintains the community in an earlier successional state.

During the early recovery period (i.e., 2010–2015) following the disturbances, the rate of recovery of coral cover differed greatly around Moorea, and contrary to expectation, coral cover returned most rapidly at sites where the least amount of live coral remained after the disturbances. Although the sites differed greatly in the rate of coral recovery, all showed at least some evidence of re-assembly to their pre-disturbance community structure in terms of relative abundance of coral genera and of the main functional groups of benthic space holders. The findings reported here suggest that, given the wide range of variation in rates of recovery and re-assembly, and the frequency of major disturbances affecting the fore reef of Moorea, not all areas are likely to attain the coral cover and taxonomic structure they had prior to the most recent disturbances before the reefs are perturbed by the next major disturbance. This is especially concerning since globally the frequency of disturbances to coral reefs is predicted to increase in the future.

## Methods

### Study site

Moorea (17°30′S, 149°50′W) is a high volcanic island with an offshore barrier reef and narrow (~0.8–1.5 km wide) lagoons (mean depth ~5–7 m) that surround its ~60 km perimeter. Between 2007 and 2009, the fore reef experienced a severe outbreak of crown-of-thorns sea stars (COTS)^23,24,26,48^, followed by a cyclone, which resulted in a decline in the cover of coral from an island-wide average of ~40% to <5% by 2010. Waves associated with the Category 4 cyclone (Cyclone Oli) that passed to the southwest of Moorea in February 2010 removed large amounts of dead coral structure from the fore reef, primarily on the north shore of the island^24^. Here we focus on the response of the fore reef benthic community to the disturbances, and the patterns and mechanisms of recovery over a five-year period following the disturbance (i.e., 2010–2015).

### Spatial and temporal patterns in abundance of benthic organisms

The Moorea Coral Reef Long Term Ecological Research project has censused the coral reef communities of Moorea since 2005, with much of the effort focused on two study sites on each of the three shores of the island (Fig. 1). Diver surveys and photoquadrats provide estimates of percent cover of major benthic substratum categories (coral, fleshy macroalgae, turf/CCA, sand, etc.), with most benthic taxa (including corals) resolved to species or genus level, thereby allowing the trajectories of disturbance effects on the community to be determined with relatively fine taxonomic resolution. Coral community structure on the fore reef is quantified using photoquadrats (0.25 m^2^) taken at 40 fixed locations located along a 50 m long transect placed along the 10 m isobath at each of six sites; photoquadrat positions were randomly selected in 2005, but thereafter the same locations have been recorded^49,50^. Photoquadrats are recorded in April in each year using a framer that holds a digital camera and two strobes (Nikonos SB105) perpendicular to, and ~1 m above, the reef. Several cameras have been used during the study, and the resolution has increased from 6 megapixels (Nikon D70), to 12 megapixels (Nikon D90), and most recently, 16 megapixels (Nikon D7000), all fitted with an 18–70 mm lens (Nikon DX). Photos are analyzed for percentage cover of benthic organisms using CPCe software^50^ using 200 randomly placed points on each image. Benthic substrata are classified in two different ways. The first quantifies cover by genus for scleractinians and *Millepora*, and the second scores cover by major functional groups – sand, macroalgae, stony coral (scleractinians and *Millepora*), and a combined category consisting of bare space, algal turf, and crustose coralline algae. Additionally, the abundance of mobile invertebrates and percent cover of genera of macroalgae along the transects at each site are estimated *in situ* by scuba divers^51^. Details concerning sampling protocols and the data can be viewed at: http://mcr.lternet.edu/data.

We used time series data describing benthic cover to explore patterns of response to, and recovery from, disturbance with a focus on spatial variation in post-disturbance recovery. Since sites reached their point of lowest coral cover in different years, and thus had different lengths of recovery time until 2015, we calculated an average annual rate of recovery to enable comparisons among them. To do this, for each photoquadrat we first identified the nadir of coral cover following the 2007–2009 COTS outbreak and the 2010 cyclone. We then took the difference between the coral cover in 2015 and the nadir, and divided it by the number of years in the ‘recovery phase’ (i.e., years since the nadir was reached). This metric (in units of % y^−1^) provided an estimate of recovery rate of coral cover at the quadrat scale, and it was used to quantify patterns of recovery among the sites. We used one-way ANOVA to test whether the return rates of coral cover varied among sites. In addition, because colonization by macroalgae can inhibit the recovery of coral communities following disturbances, we used linear regression to test whether variation in the mean cover of macroalgae during the recovery period was associated with variation in coral recovery rates among sites.

### Recruitment, growth, and survivorship of coral

Our analyses demonstrated that the early period of recovery of the coral community following the COTs outbreak and Cyclone Oli largely was driven by *Pocillopora* spp. (Supplementary Fig. S3; see^27^), and therefore we focused our demographic studies on this genus. With respect to scleractinians, ‘recruits’ have been defined in multiple ways in previous studies, ranging from the smallest corals (a few mm in size) that can be detected on natural reef surfaces or settlement tiles, and which probably are only a few weeks or months old, to larger colonies that are likely to be many months or several years in age. We operationally defined recruits as colonies ≤3.0 cm diameter, recognizing that they represent corals varying in age, but that likely arrived on the reef during the previous 12 months (i.e., between annual sampling). On the fore reef of Moorea, *Pocillopora* is represented by at least seven species that are difficult to resolve by gross morphology: *P. verrucosa, P. meandrina, P. woodjonesi, P. eydouxi, P. effusus*, and two un-named haplotypes^52^. *P. damicornis* is also found in Moorea, but it is uncommon on the fore reef. Given the challenges of distinguishing among *Pocillopora* species, especially when colonies are very small, it is highly likely that the *Pocillopora* recruits we enumerated reflect multiple species.

We estimated recruitment of *Pocillopora* using two techniques that quantified the occurrence of young colonies on natural reef surfaces. The first was diver surveys that enumerated recruits of *Pocillopora* (as well as *Porites* and *Acropora*) in 5 × 5 m plots (25-m^2^ plots; N = 5–20 plots site^−1^, N = 45 total), annually during the Austral winter from 2010 to 2015. Plots were randomly located and permanently marked, and were situated at 10 to 12 m depth and ≥10 m apart. Divers searched each plot and recorded the number and sizes (length, width) of coral colonies in the three genera. Surveys of these plots enabled estimation of recruitment over relatively large areas on the reef. The second method measured recruitment on a smaller spatial scale, using the 0.5 × 0.5 m photoquadrats (0.25-m^2^ photoquadrats; N = 40 site^−1^) recorded annually at all sites. The number of *Pocillopora* spp. recruits in each photoquadrat from 2011 to 2014 was counted. Corals were only counted as recruits if they were ≤3 cm diameter and they were not observed in the photoquadrat in the previous year. Measurements of recruitment from diver surveys and photoquadrats were positively correlated (pooled among all sampling years, r = 0.91, P = 0.01; Supplementary Fig. S5).

The photoquadrats were also used to evaluate the growth and survivorship of *Pocillopora* spp. recruits for the first year following their detection in the photoquadrats. To estimate annual survivorship, photoquadrats were compared between consecutive years (2011 and 2012, 2012 and 2013, and 2013 and 2014) and *Pocillopora* spp. recruits in each photoquadrat tracked from one year to the next, and scored for survivorship (alive in both years) and growth (change in diameter). Sampling prior to 2011 was not included as the objective was to evaluate processes supporting recovery of coral community structure following the COTS outbreak and Cyclone Oli in 2010. From these data we calculated mean survival and growth of recruits for each photoquadrat; these means were used in subsequent analyses of growth and survivorship of *Pocillopora* spp. We used one-way ANOVAs to test whether each of the demographic rates (e.g., recruitment, recruit growth, and recruit mortality) varied among sites. We also report the percent of variation in each demographic rate that can be attributed to each of the spatial scales of observation (i.e., site, 25-m^2^ plot, and 0.25-m^2^ photoquadrat) since this could provide insight into the potential mechanisms driving variation in demographic rates.

### Spatial variation in patterns of disturbance and recovery

To better understand the mechanisms responsible for the patterns of coral recovery, we tested the relative importance of three demographic rates (e.g., recruitment, recruit growth, and recruit mortality) in driving spatial patterns of recovery of *Pocillopora* cover. These analyses were conducted at two different spatial scales (site and photoquadrats). To test whether variability in recovery rates among sites was associated with site-level variation in demographic rates, we first calculated mean values for each demographic rate by site. We then used three linear regressions to test separately whether any of the demographic rates could predict recovery at the site scale. In addition to these site-scale analyses, we also tested whether the early demography of *Pocillopora* could explain variation in recovery at the scale of photoquadrats. While we were interested in explaining variation in recovery rates among sites, photoquadrat-level analyses provided additional insight into the way in which variation in demographic rates was associated with recovery.

To determine if early demography of *Pocillopora* could explain variation in recovery at the photoquadrat scale, we compared a series of general linear models with combinations of four numerical predictors and one categorical predictor: site, cumulative recruitment (number of new recruits each year, summed over 2011–2014), the nadir of *Pocillopora* spp. cover, mean recruit growth (mm y^−1^), and mean annual recruit survivorship (%). Candidate models were chosen by starting with a full model, and then using backward stepwise regression to sequentially remove factors one at a time based on their F-ratios. Candidate models were compared with likelihood ratio tests^53^ in R 3.3.2^54^. After identifying the best model, we calculated partial R squares for each predictor variable using the ‘lmg’ metric in the R package relaimpo^55^ following the method of Chevan and Sutherland^56^. The photoquadrat-level analysis necessarily involved a truncated data set (because estimates of recruit growth rates and mortality rates could not be obtained for quadrats in which recruits were not found). Thus, we also ran the same analysis (excluding recruit growth and recruit mortality as predictors) using the full data set for comparison; these results were nearly identical and are not reported.

NMDS analysis of trajectories of three aspects of reef community structure (functional groups, coral taxa, algal taxa) from 2005 to 2015 were used to depict temporal patterns of these aspects of community structure for three sites (one on each side of the island), representing the range of community recoveries observed during the study. The functional group analysis was based on four categories of benthic cover: scleractinian corals (plus *Millepora*), macroalgae, sand, and a combined group of turf, crustose coralline algae, and bare space. The analysis of macroalgal taxa was based on estimates of percent cover, largely at the level of genus and species (14 groups: *Amansia rhodantha*, *Amphiroa fragilissim*a, *Asparagopsis taxiformis*, *Caulerpa* spp., *Cladophoropsis membranacea*, *Codium geppiorum*, Cyanophyta, *Dictyota* spp., *Halimeda* spp., *Jania* sp., *Lobophora variegata*, *Peyssonnelia* spp., *Symploca hydnoides*, *Turbinaria ornata*). Analyses of corals were based on percent cover by genus and family (18 groups: *Acanthastrea*, *Acropora*, *Astreopora*, *Cyphastrea*, *Dipsastraea*, Fungiidae, *Gardineroseris*, *Leptastrea*, *Leptoseris*, *Lobophyllia*, *Millepor*a, *Phymastrea*, *Montipora*, *Pavona*, *Pocillopora*, *Porites*, *Psammocora*, *Stylocoeniella*). Data were square root (functional groups), log(x + 1) (corals) or fourth root (algae) transformed prior to preparing resemblance matrices by Bray-Curtis dissimilarity. These analyses provided insight on the magnitude of the effects the disturbances on community structure, as well as the pace of recovery to the initial (pre-disturbance) community state. Plots of the Student t statistic from multiple contrasts (in PERMANOVA) of the similarity values over time between community composition each year compared to the initial year of the time series (2005) revealed the degree to which sites approached their pre-disturbance community structure. Analyses were conducted separately by site using PERMANOVA with years and time as factors in a repeated measures design with quadrats as replicates. These analyses were done using PRIMER^57^ and PERMANOVA+^58^.

Permits for field work were issued by the Haut-commissariat de la République en Polynésie Française (DRRT) (Protocole d’Accueil 2005–06, 2006–07, 2007–08, 2008–09, 2009–10, 2010–11, 2011–12, 2012–13, 2013–14 and 2014–15 to RJS, SJH, RCC and PJE) for research associated with the US National Science Foundation Moorea Coral Reef Long Term Ecological Project.

Data for this study are available on the website of the Moorea Coral Reef Long Term Ecological Project: http://mcr.lternet.edu/data^49,51^.

## Electronic supplementary material

Supplementary Materials
