# Supplementary material for: Recruitment Drives Spatial Variation in Recovery Rates of Resilient Coral Reefs
Source: Sci Rep. 2018 May 9;8:7338. doi: 10.1038/s41598-018-25414-8 (PMC5943288; doi:10.1038/s41598-018-25414-8)
Supplement: Supplementary file 1 — Supplementary Materials [file 41598_2018_25414_MOESM1_ESM.pdf]

Supplementary Materials

Recruitment Drives Spatial Variation in Recovery Rates of Resilient Coral Reefs

Sally J. Holbrook[1,2*], Thomas C. Adam[2], Peter J. Edmunds[3], Russell J. Schmitt[1,2], Robert C. Carpenter[3], Andrew J. Brooks[2], Hunter S. Lenihan[2,4] and Cheryl J. Briggs[1]

[1]Department of Ecology, Evolution and Marine Biology, University of California Santa Barbara, Santa Barbara CA 93106 USA

[2]Coastal Research Center, Marine Science Institute, University of California Santa Barbara, Santa Barbara CA 93106 USA

[3]Department of Biology, California State University Northridge, Northridge CA 91330 USA

[4]Bren School of Environmental Science and Management, University of California Santa Barbara, Santa Barbara CA 93106 USA

Table S1. Relative abundance of major coral genera in the pre- and post-disturbance time periods (2005 and 2015, respectively).

	Acropora		*Montipora*		*Pocillopora*		*Porites*		Other Scleractinians	
Site	2005	2015	2005	2015	2005	2015	2005	2015	2005	2015
LTER 1	33.2	3.6	0.6	15.1	40.6	70.1	21.4	7.3	4.1	3.8
LTER 2	32.9	2.5	2.0	12.1	36.5	67.7	24.2	13.4	4.4	4.2
LTER 3	28.4	4.3	2.0	13.8	45.7	49.7	9.9	15.4	14.0	16.8
LTER 4	8.1	5.5	16.3	35.1	65.6	39.7	5.0	6.7	5.0	13.1
LTER 5	10.1	6.4	17.8	26.8	46.3	41.3	16.7	16.7	9.1	8.7
LTER 6	18.2	3.8	9.5	16.5	57.2	56.5	6.7	11.5	8.4	11.7

Table S2. Results of model selection testing the effects of site, cumulative recruitment, minimum cover of *Pocillopora* spp., mean recruit growth, and mean recruit survivorship on recovery rate. Models were compared using likelihood ratio tests. P values compare the model in a given row with the model above it. Results indicate that the best model included effects of cumulative recruitment, minimum cover of *Pocillopora* spp., and mean recruit growth, but not mean recruit survivorship.

**Model**	**Residual df**	**Residual SS**	**df**	**P**
Site (S)	143	608.46		
S + Cumulative recruitment (CR)	142	538.92	1	0.00001***
S + CR + Minimum *Pocillopora* (MP)	141	512.14	1	0.006**
S + CR + MP + Mean recruit growth (G)	140	496.64	1	0.04*
S + CR + MP + G + Mean recruit survivorship	139	496.55	1	0.87

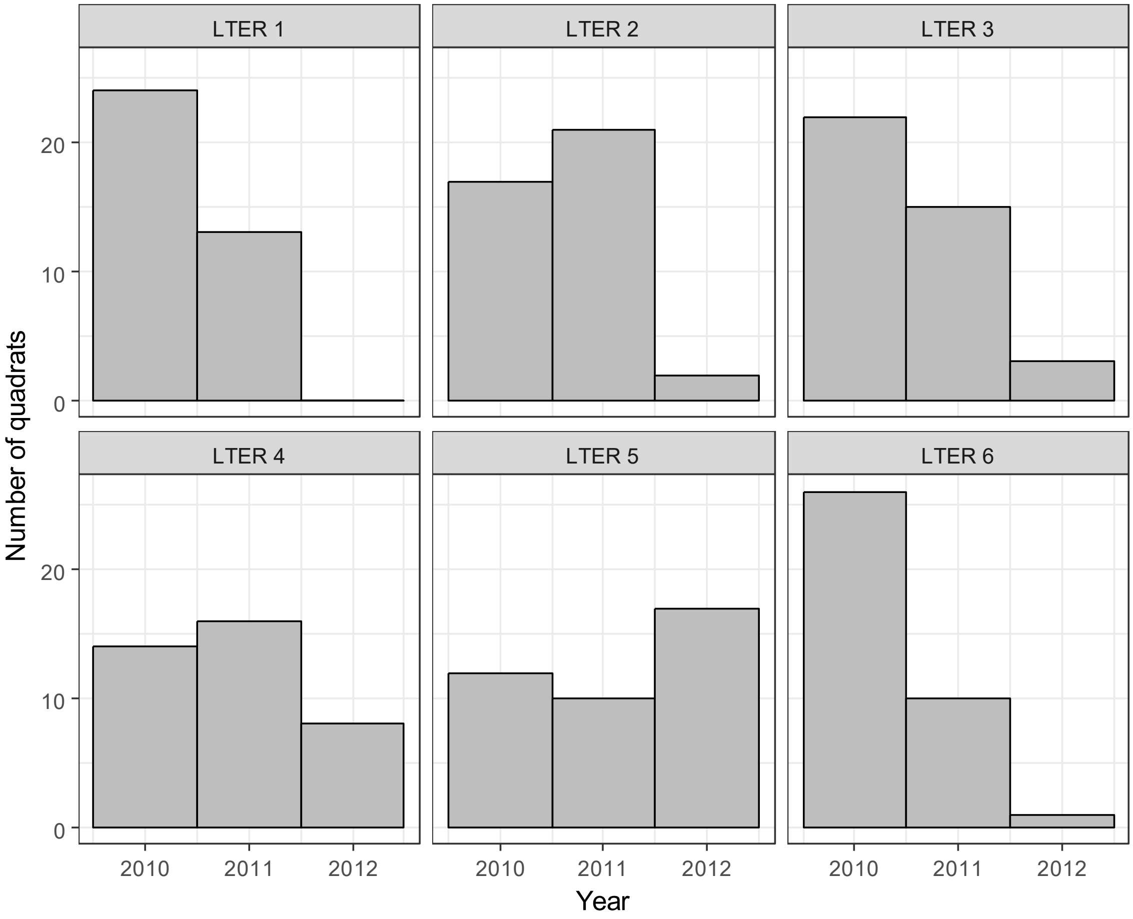

Figure S1. Histograms showing the frequency distribution of the year (2010, 2011, or 2012) in which the 0.25-m^2 photoquadrats reached minimum cover of coral. The counts represent the number of quadrats that reached their nadir in coral cover in each year. Data for the six fore reef sites on Moorea are shown separately.

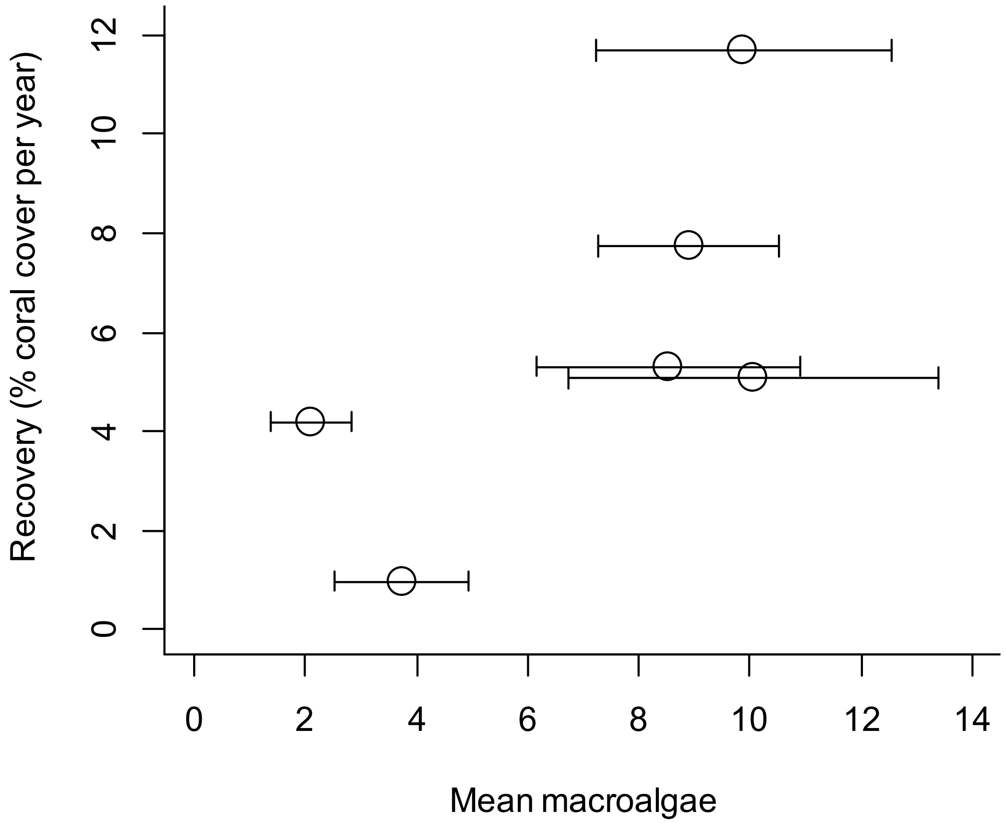

Figure S2. The relationship between the mean percent cover of macroalgae during the recovery period (2010-2015) and the subsequent recovery rate (i.e., percent coral cover year $^{-1}$) at the six fore reef study sites. The time averaged cover of macroalgae remained < 15% on all of the sites during the recovery period, and an ANOVA that tested the significance of the regression model of the relationship between the rate of increase in coral cover and the time-averaged cover of macroalgae was not significant ($F_{1,4}$ = 3.34, P = 0.14). Error bars are +/- 1 SE (n = 6 sampling years).

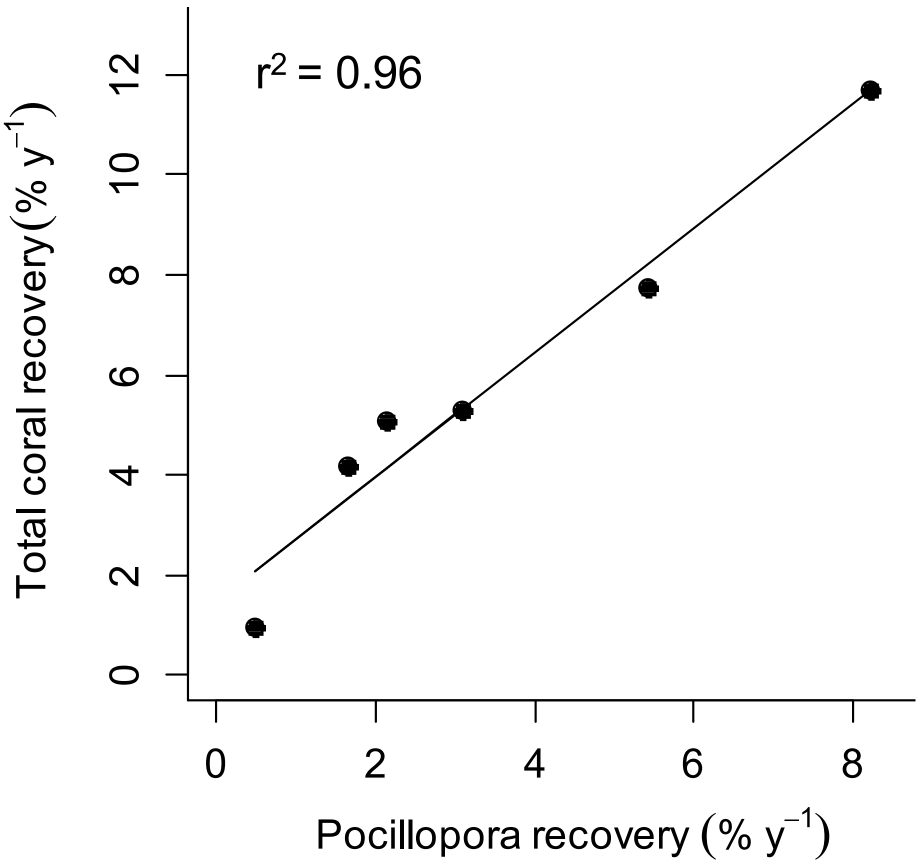

Figure S3. Relationship between the rate of recovery of *Pocillopora* spp. cover and the rate of recovery of total coral cover ($F_{1,4}$ = 88.56, P = 0.0007) at the six fore reef sites.

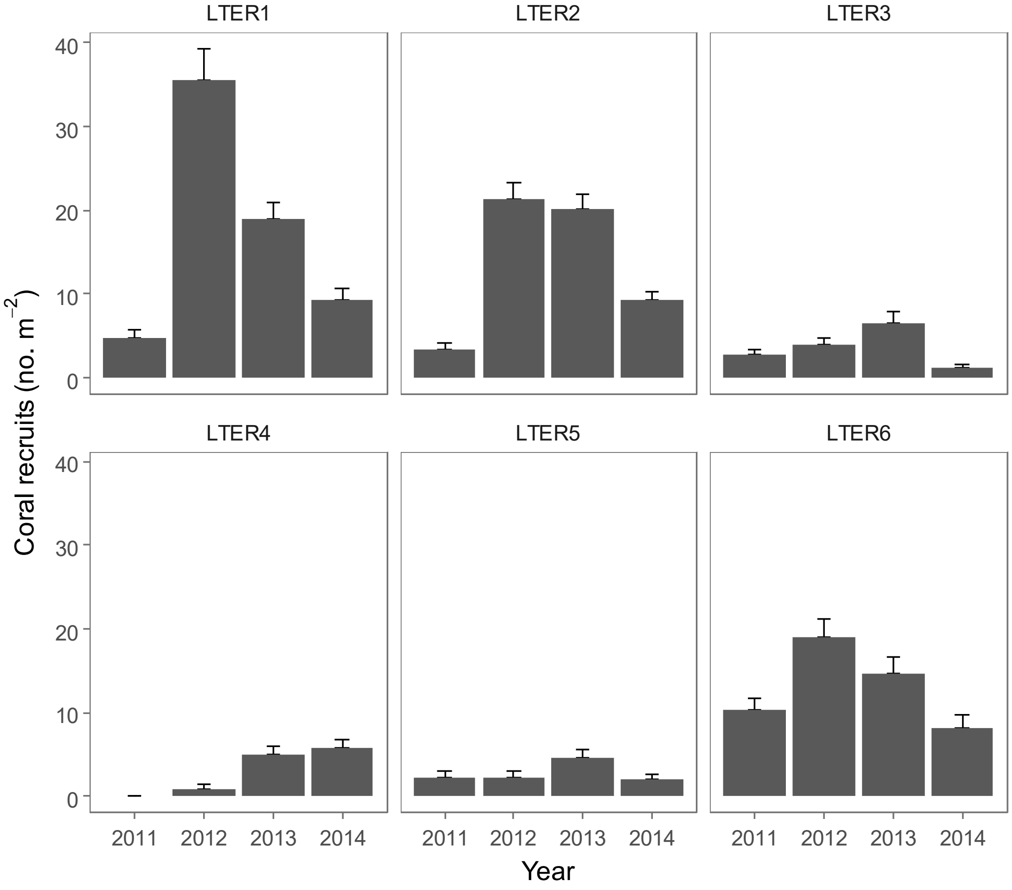

Figure S4. Coral recruitment observed in 0.25-m^2 photoquadrats. Shown are mean (+SE) density of *Pocillopora* recruits detected in photoquadrats at each fore reef site during 2011 to 2014. The total number of *Pocillopora* recruits observed on the photoquadrats over the time period at the six sites = 1893.

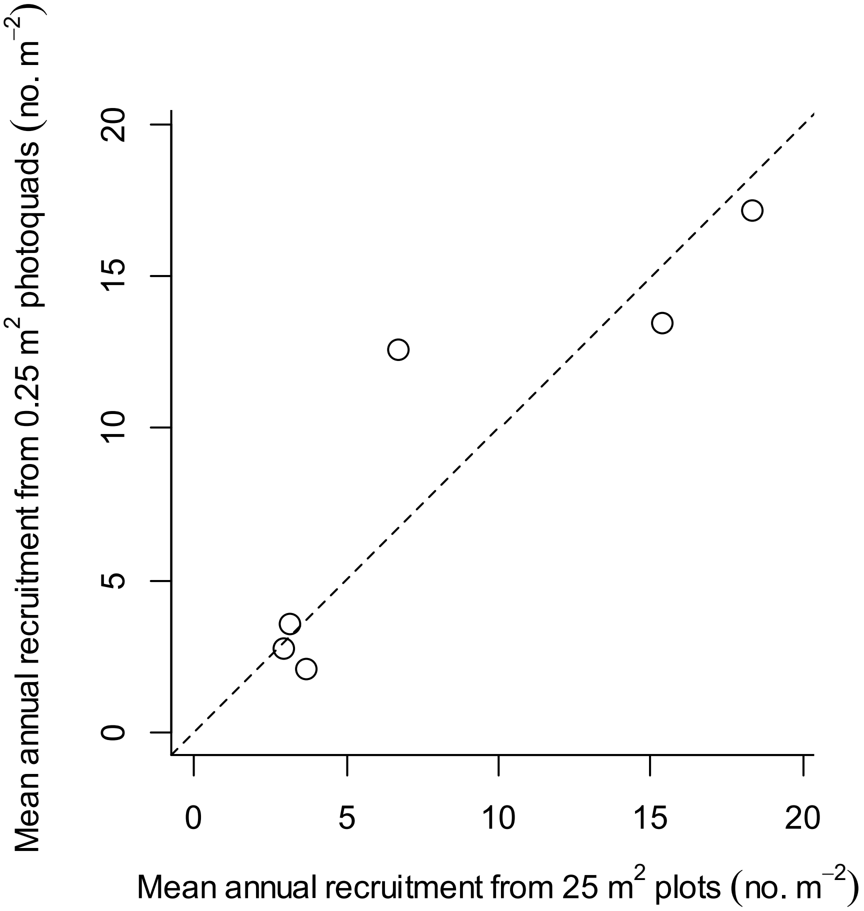

Figure S5. Correlation between two different estimates of *Pocillopora* recruitment – in situ diver surveys on 25-m^2 plots (x axis) and analysis of 0.25-m^2 photoquadrats (y axis). Dotted line is the 1:1 line. Dots represent site means from 2011 to 2014. Estimates from the two methods are significantly positively correlated ($r = 0.91$, $P = 0.01$) at the site scale.
